# Pulmonary infection due to fluoroquinolone-resistant *Mycolicibacterium fortuitum*: a case report

**DOI:** 10.1186/s12879-020-05596-1

**Published:** 2020-11-19

**Authors:** Kana Kurokawa, Norihiro Harada, Hitoshi Sasano, Haruhi Takagi, Satomi Takei, Ayako Nakamura, Keisuke Kamada, Atsushi Yoshida, Ken Kikuchi, Kazuhisa Takahashi

**Affiliations:** 1 Department of Respiratory Medicine, Juntendo University Faculty of Medicine and Graduate School of Medicine, 3-1-3 Hongo, Bunkyo-ku, Tokyo, 113-8431 Japan; 2 Department of Clinical Laboratory, Juntendo University Hospital, 3-1-3 Hongo, Bunkyo-ku, Tokyo, 113-8431 Japan; 3 Department of Clinical Laboratory, Juntendo Tokyo Koto Geriatric Medical Center, 3-1-3 Hongo, Bunkyo-ku, Tokyo, 113-8431 Japan; 4 Department of Infectious Diseases, Tokyo Women’s Medical University, 8-1 Kawada-cho, Shinjuku-ku, Tokyo, 162-8666 Japan

**Keywords:** *Mycolicibacterium fortuitum*, Fluoroquinolone, Resistance, DNA gyrase, *gyrA*

## Abstract

**Background:**

*Mycolicibacterium fortuitum* is a species of the rapidly growing mycobacteria that can cause pulmonary infection. It is susceptible to multiple antibiotics both in vitro and in clinical practice, so that any combination of susceptible drugs is effective. However, we encountered a case of infection due to fluoroquinolone-resistant *M. fortuitum*. In this study, we report the case and describe the mechanism of resistance.

**Case presentation:**

A 65-year-old man with a history of total gastrectomy and immunosuppressant treatment for rheumatoid arthritis developed a recurrence of pulmonary infection caused by *M. fortuitum*. He was treated with clarithromycin and levofloxacin as a first-line treatment, based on the favorable susceptibility at that time. After recurrence, a high minimum inhibitory concentration to fluoroquinolones was detected. DNA sequencing of the pathogen showed the substitution of serine for tryptophan at residue 83 in the *gyrA* gene. He was successfully treated with a combination of other antibiotics.

**Conclusion:**

This is the first report on the treatment of fluoroquinolone-resistant *M. fortuitum* and investigation of the mechanism of resistance. We suggest that the susceptibility test remains effective for determining the next line of treatment after a pathogen has acquired resistance, and resistance to fluoroquinolones in *M. fortuitum* can be attributed to a single change of amino acid.

## Background

Non-tuberculous mycobacteria (NTM) are widespread in the natural environment, including natural waters, engineered water systems, and soils [1]. They can affect many organs and cause diseases, such as pulmonary disease, lymphadenitis, cutaneous disease, and disseminated disease [2]. Of these, pulmonary infection is the most common clinical manifestation [2]. The most common pathogens for lung disease are *Mycobacterium avium complex* and *Mycobacteroides abscessus*, but *Mycolicibacterium fortuitum* is also important [3]. *M. fortuitum* is one of the rapidly growing mycobacteria (RGM), which is the categorization according to colony morphology and growth characteristics [2]. One characteristic of *M. fortuitum* is to show favorable susceptibility and clinical effect of multiple antibiotics [4]. The 2007 official American Thoracic Society (ATS)/Infectious Diseases Society of America (IDSA) statement showed that *M. fortuitum* isolates were susceptible to amikacin (100%), ciprofloxacin and ofloxacin (100%), sulfonamides (100%), cefoxitin (50%), imipenem (100%), clarithromycin (80%), and doxycycline (50%) [4]. Several previous cases have reported that combining antibiotics, including quinolones, successfully completed the treatment [5, 6]. There is no report about the treatment of resistant *M. fortuitum.*

Quinolones act by inhibiting the bacterial topoisomerases DNA gyrase and topoisomerase IV [7]. The most common mechanism of quinolone resistance in mycobacteria is due to mutations in the *gyrA* and *gryB* genes of DNA gyrase [7]. These mutations involved a conserved region called the quinolone resistance-determining regions (QRDR) [7]. However, there have been no reports on *M. fortuitum* and its mechanism of resistance.

We describe a rare case of pulmonary disease due to fluoroquinolone-resistant *M. fortuitum.* In addition, we investigate the mechanism of resistance of *M. fortuitum*.

## Case presentation

A 65-year-old man (height 166.1 cm, weight 63.0 kg) with a history of total gastrectomy for gastric cancer was admitted to our hospital with complaints of cough, sputum, and fever. At 61 years of age, he was diagnosed with rheumatoid arthritis. He underwent surgical lung biopsy for evaluation of a 3-year history of unchanged reticular shadow observed in his chest. Histopathological analysis showed uniform involvement of alveolar wall fibrosis with lymphoid follicles, which was consistent with a diagnosis of collagen vascular disease-associated interstitial pneumonia (CVD-IP). Tacrolimus (1 mg/day) and salazosulfapyridine (1000 mg/day) were started to treat the rheumatoid arthritis.

**Fig. 1:**
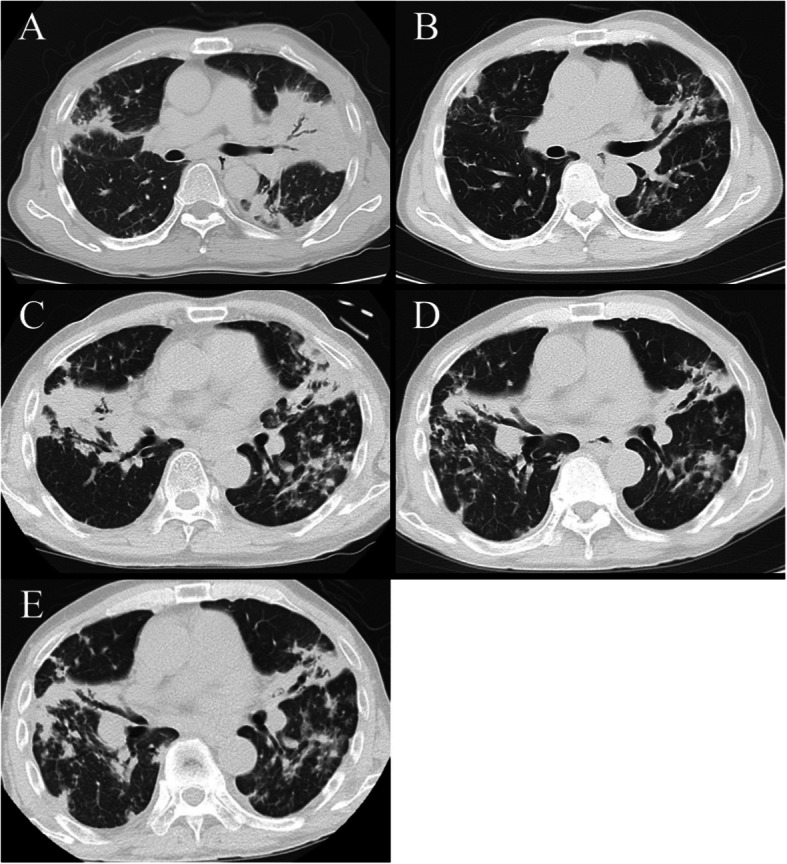
Chest computed tomography. **a** Computed tomography showed consolidations in the right middle and left upper lobe when *Mycolicibacterium fortuitum* was detected for the first time. **b** After treatment with clarithromycin and levofloxacin, consolidation improved. **c** Bilateral lung consolidation occurred. **d** After intravenous antibiotic therapy, pulmonary shadow improved. **e** After discharged and treated for 6 months as an outpatient

At 63 years of age, we found pulmonary consolidation in the left middle and lower lung field on chest radiograph and was more striking, especially in left upper lobe on computed tomography (Fig. 1a), despite adding 30 mg of prednisolone (PSL) for the progression of CVD-IP. Bronchoscopy was performed for detecting pathogen, and the microscopic examinations of sputum and bronchial lavage smear stained with the Ziehl-Neelsen staining technique scored a grade of 2 respectively. Moreover, *M. fortuitum* identified by matrix-assisted laser-desorption/ionization time-of-flight mass spectrometry was isolated from the bronchial lavage specimen. Susceptibility testing was performed according to Clinical and Laboratory Standard Institute guideline M24 3rd Edition [8], using a broth microdilution method and cation-adjusted Mueller-Hinton broth. We prescribed clarithromycin (600 mg/day) and levofloxacin (500 mg/day) based on the result of susceptibility tests (Table 1). The dose of clarithromycin was determined considering the efficacy and the risk of gastrointestinal toxicity because he had esophageal reflux after total gastrectomy. The consolidations improved gradually (Fig. 1b). Antibiotic treatment was discontinued 12 months after sputum cultures yielded negative results, and the dosage of PSL for CVD-IP was tapered to 15 mg.

**Table 1:** Antimicrobial agents and MIC breakpoints for rapidly growing mycobacteria

	MIC (μg/mL) for category			*before* treatment	*after* treatment
	Susceptible	Intermediate	Resitant		
Amikacin	≤16	32	≥64	< 1 (S)	4 (S)
Cefoxitin	≤16	32–64	≥128	32 (I)	6432 (I)
Ciprofloxacin	≤1	2	≥4	0.25 (S)	832 (R)
Clarithromycin	≤2	4	≥8	2 (> 32)^a^ (R)	8 (> 32)^a^ (R)
Imipenem	≤4	8–16	≥32	2 (S)	4 (S)
Linezolid	≤8	16	≥32	8 (S)	8 (S)
Meropenem	≦4	8–16	≧32	2 (S)	4 (S)
Moxifloxacin	≤1	2	≥4	0.25 (S)	8
Trimethoprim-sulfamethozazole	≤2/38	–	≥4/76	0.5/9.5 (S)	0.5/9.5 (S)
Tobramycin	≤2	4	≥8	16 (R)	32 (R)
Levofloxacin^b^	≤1	2	≥4	0.5 (S)	8 (R)
Sitafloxacin^c^				0.25	1

The drug susceptibility test was performed in accordance with the standard operation procedures of the Clinical and Laboratory Standard Institute M24A-3

*Abbreviations*: *after treatment* after treatment with clarithromycin and levofloxacin, *before treatment* before treatment with clarithromycin and levofloxacin, *I* intermediate, *MIC* minimum inhibitory concentration, *R* resistant, *S* susceptible

^a^MIC after 3 days incubation priod (in parentheses, 14 days incubation priod)

^b^the MIC breakpoints of ciprofloxacin and levofloxacin are interchangeable

^c^reference value

However, only 1 month after the end of the antibiotic treatment, pulmonary consolidation in the left lung and middle lung field worsened again, and new consolidation occurred in the right middle lung field. Despite increasing PSL to 40 mg and re-administration of clarithromycin and levofloxacin, his cough and sputum worsened, and his body temperature persisted over 38 °C. Six months after the end of the initial antibiotic treatment, we admitted him for further examinations and intravenous antibiotic therapy. On admission, he was ill-appearing and febrile to 37.5 °C with an oxygen saturation of 96% on room air. His chest auscultation revealed coarse crackles over the inferior zone of the lungs. Laboratory examinations revealed an increased white blood cell count of 15,900/μL and elevated C-reactive protein level 4.45 mg/dL. Sialylated carbohydrate antigen KL-6, surfactant protein-D, anti-cyclic citrullinated peptide antibody, and matrix metalloproteinase-3 were 803 U/mL, 112 mg/mL, 14.4 U/mL, and 791.7 mg/mL, respectively. These levels of serum markers for interstitial pneumonia and rheumatoid arthritis were high but remained unchanged for several months. A chest radiograph showed that consolidation was widespread. A chest computed tomography scan showed consolidations in the bilateral upper and middle lobe predominance (Fig. 1c). Although we confirmed Ziehl-Neelsen stained smear samples of sputum and bronchial lavage scored the grade of 2 and detected *M. fortuitum* from both sputum culture and bronchial lavage culture, its susceptibility differed from the previous one. It demonstrated minimum inhibitory concentration (MIC) > 4 mg/L to ciprofloxacin and levofloxacin (Table 1). Intravenous administrations of amikacin (900 mg/three-times-weekly) and imipenem/cilastatin (1500 mg/day), and oral administrations of clarithromycin and sitafloxacin (100 mg/day) were started based on the result of antimicrobial susceptibility testing. His symptoms and chest imaging improved gradually (Fig. 1d and e). The intravenous imipenem/cilastatin treatment course was completed, and he was discharged four weeks after administration. In an outpatient setting, clarithromycin, sitafloxacin, and three-times-weekly amikacin were continued. Moreover, faropenem (600 mg/day) and trimethoprim-sulfamethoxazole (trimethoprim 320 mg/day and sulfamethoxazole 1600 mg/day) were added after discharge, despite faropenem has no certain evidence of efficacy for *M. fortuitum*. Because we have very limited options for antibiotics to replace imipenem infusions in Japan. Although amikacin was discontinued after 11 months because auditory dysfunction appeared, not only his symptoms for infection but also the consolidation in chest x-ray did not get worse for 10 months after amikacin discontinuation. Over time, his PSL gradually diminished as his arthritis symptoms also improved.

**Fig. 2:**
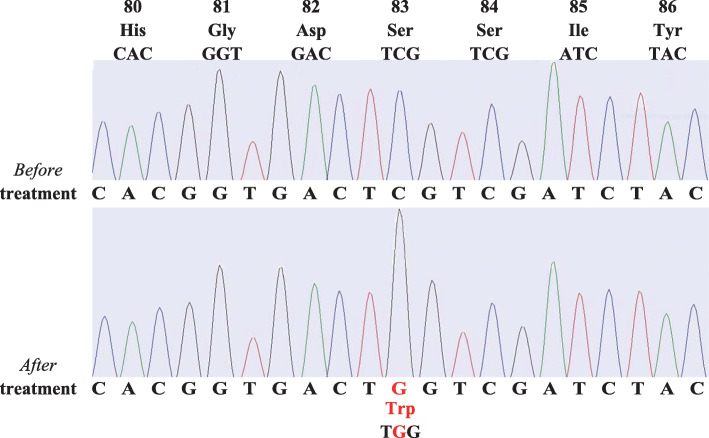
Analysis of fluoroquinolone resistance determining region in *gyrA*. At position 83, the amino acid was serine before treatment with clarithromycin and levofloxacin (*before* treatment). After these treatment (*after* treatment), serine converted to tryptophan

The DNA gyrase gene of *M. fortuitum* was sequenced. Chromosomal DNA was extracted by the freezing-boiling technique of Woods and Cole, as previously described [9]. DNA fragments corresponding to the QRDR of GyrA were amplified by polymerase chain reaction using previously described primers Pri9 (5′-CGCCGCGTGCTGCATGCAGATG-3′) and Pri8 (5′-CTGGTGGAGTCAGT TA/GCCC/TGGCGA-3′) [10]. We tested *M. fortuitum* DNA gyrase from both before and after the treatment with clarithromycin and levofloxacin. The amino acid at residue 83 of the *gyrA* gene changed from serine to tryptophan (Fig. 2 and Table 2).

**Table 2:** Alignment of the peptide sequences of the QRDR of GyrA

reference	ARSVAETMGNYHPHGD **S** SIYDTLVRMAQPWSLRYPLVDGQ
*before* treatment	ARSVAETMGNYHPHGD **S** SIYDTLVRMAQPWSLRYPLVDGQ
*after* treatment	ARSVAETMGNYHPHGD **W** SIYDTLVRMAQPWSLRYPLVDGQ

The GyrA QRDR extends from the amino acid residues 67 to 106 in the numbering system used for *E. coli*. biov., biovariant. They were identical for all species, except for the amino acid residue at position 83

*Abbreviations*: *after treatment* after treatment with clarithromycin and levofloxacin, *before treatment* before treatment with clarithromycin and levofloxacin, *QRDR* Quinolone resistance determining region

## Discussion and conclusions

To our knowledge, this is the first report to describe the treatment of fluoroquinolone-resistant *M. fortuitum* and identify the mechanism. *M. fortuitum* is one of RGM [4], commonly seen as an infection through whirlpool footbaths in nail salons [11–14]. It most commonly causes localized infections of the skin, bone, and soft tissue disease [4]. While pulmonary disease due to *M. fortuitum* is relatively rare [3], some risk factors have been reported. Related structural lung diseases such as chronic obstructive pulmonary disease and bronchiectasis, neoplasms, immunosuppressant use, and environmental exposure are known to be risk factors in other NTM-related pulmonary diseases [15]. Gastrointestinal disturbance with chronic vomiting is also a characteristic predisposing factor for *M. fortuitum* [4]. Our patient had many risk factors including current use of an immunosuppressant, previously treated neoplasms, and having a gastrointestinal disorder secondary to a total gastrectomy.

Treatment of NTM is based on their unique susceptibility patterns and sensitivities to antibiotics [4]. *M. fortuitum* is one of the most susceptible groups in vitro, which closely corresponds to the clinical effect. Therefore, using at least two antibiotics based on susceptibility test is recommended and the duration of treatment should be given for at least 12 months after negative sputum culture is obtained [4]. Any two-drug combination with in vitro susceptibility should be successful [4]. In particular, resistance to fluoroquinolones is quite rare [16–19]; therefore, fluoroquinolones are recommended as a treatment option for *M. fortuitum* [16]. Since it is rare to acquire resistance in *M. fortuitum*, there is no report on treating it. In our case, the result of susceptibility test in vitro even after acquiring resistance was also parallel to those of clinical effect. The antibiotics which showed high MIC became clinically ineffective. Moreover, treatment with amikacin and imipenem/cilastatin succeeded, as susceptibility test indicated. Therefore, we suggest that susceptibility test is still a valid metric to choose antibiotics in patients who have been previously treated.

As far as we know, the mechanism of resistance to fluoroquinolones in *M. fortuitum* has never been reported. Quinolones inhibit both bacterial DNA gyrase and topoisomerase IV in other bacteria, whereas DNA gyrase is the only target in mycobacteria [7]. This enzyme is a tetrameric protein composed of two A and two B subunits, carrying *gyrA* and *gyrB* gene products respectively [7]. One mechanism of resistance is associated with a difference in the structures of subunits, including only a single amino acid change in these genes [20]. Several amino acids are reported to be related to quinolone resistance, which commonly localizes in the limited regions, called the QRDR [20]. Using in the numbering system used for *Escherichia coli*, the residue at position 83 in the QRDR GyrA plays an important role [20]. Although most quinolone-resistant species such as *M. abscessus* or *M. avium* have an alanine residue at position 83 in GyrA, wild-type *M. fortuitum* GyrA has a serine which is the same structure as quinolone-susceptible bacteria such as *E. coli* and *Neisseria gonorrhoeae* [7]. In our case, a strain before antibiotic treatment had a serine residue at position 83 in the A subunit of DNA gyrase, as same as wild-type *M. fortuitum*. However, after treatment with clarithromycin and levofloxacin, a serine was substituted by a tryptophan. We hypothesize that the conversion of a small polar amino acid to a large hydrophobic residue leads to quinolone resistance in this case. This hypothesis is supported by an analysis of *E. coli* GyrA, which showed that this change can cause quinolone resistance [21]. Unfortunately, in our case, it was difficult to accurately distinguish between recurrence due to acquisition of quinolone resistance and reinfection of quinolone-resistant strain because no molecular typing tests were performed. However, the infection was suspected to have recurred because it worsened just 1 month after the antibiotics were discontinued.

Although a certain breakpoint has not been established for sitafloxacin, *M. fortuitum* was reported to have low MIC to sitafloxacin [16]. Recent studies showed that ciprofloxacin or levofloxacin-resistant strains with GyrA mutations remain low MIC to sitafloxacin in *N. gonorrhoeae*, *Mycobacterium tuberculosis*, and *M. avium complex* [22–24]. Whereas study in *Helicobacter pylori* infection after unsuccessful eradication with sitafloxacin-containing regimens showed that sitafloxacin might lead to the accumulation of double mutations in GyrA, which would provide resistance to sitafloxacin in *Helicobacter pylori* [25]. The present case also showed low MIC to sitafloxacin in strains with ciprofloxacin and levofloxacin-resistance (Table 1). Further study is needed for the investigation of that mechanism and clinical efficacy of sitafloxacin, as it could be a candidate as a suitable option because a variety of oral treatment for RGM is limited.

The official statement by ATS/IDSA in 2007 recommended that clarithromycin for RGM should be used with caution [4]. It is mainly because of the presence of the erythromycin-inducible methylase (*erm*) gene, which methylates the 23S ribosomal RNA macrolide-binding site, well-known as a common cause of inducible resistance to macrolides [26]. Some RGM species have their own intrinsic *erm* gene, such as the *erm* (39) genes of *M. fortuitum* or the *erm* (41) genes of *M. abscessus*. Due to the *erm* gene, isolates became resistant to clarithromycin with 14 days of incubation, although these showed the susceptible MICs at 3 days of incubation [27]. Our case showed susceptibility at day 3, but resistance at day 14 to clarithromycin (Table 1). Because of limited choices of oral antibiotics and the potency of these combinations of antibiotics, we did not discontinue clarithromycin. Although how to use clarithromycin for RGM is still controversial, further studies are required to evaluate the effect of combination therapy to increase options for the treatment of RGM.

In conclusion, this is the first report to describe the treatment of a pulmonary infection due to fluoroquinolone-resistant *M. fortuitum*. Although it has been reported as one of treatable species in mycobacterium, it can become resistant to fluoroquinolones. Susceptibility test is still effective over second-line treatment. Moreover, a single amino acid substitution in DNA gyrases can lead to resistance, even in *M. fortuitum*.

## Data Availability

Data sharing is not applicable to this article as no datasets were generated or analyzed during this case report.

## Abbreviations

CVP-IP: Collagen vascular disease-associated interstitial pneumonia
*erm*: Erythromycin-inducible methylase
MIC: Minimum inhibitory concentration
NTM: Non-tuberculous mycobacteria
PSL: Prednisolone
QRDR: Quinolone resistance determining region
RGM: Rapid growing mycobacteria
